# Long-term effectiveness of a primary care chronic disease management program in Korea

**DOI:** 10.4178/epih.e2026023

**Published:** 2026-05-27

**Authors:** Seo-Hyun Lee, Kyoung-Hoon Kim

**Affiliations:** Department of Health Administration, Kongju National University College of Nursing and Health, Gongju, Korea

**Keywords:** Diabetes mellitus, type 2, Primary care, Preventable admission, Complications

## Abstract

**OBJECTIVES:**

This study evaluated the long-term effectiveness of Korea’s Primary Care Chronic Disease Management Program (CDMP) on major clinical outcomes among patients with type 2 diabetes mellitus (T2DM).

**METHODS:**

Patients aged ≥18 years who received outpatient care for T2DM in 2019 were identified using Korea’s National Health Insurance Claims Database. Patients with a prior history of inpatient or outpatient care for ischemic heart disease (IHD) or stroke, or a history of hospitalization for diabetes before the index date were excluded. The final cohort included 1,817,682 patients, who were followed from the index date to December 31, 2023, for preventable diabetes-related hospitalization and incident IHD and stroke. Cox proportional hazards models were used to estimate hazard ratios (HRs) and 95% confidence intervals (CIs).

**RESULTS:**

The study included 49,449 CDMP participants and 1,768,233 non-participants. The mean ages of the CDMP and non-CDMP groups were 62.2 years and 61.5 years, respectively. Compared with non-participants, CDMP participants had significantly lower risks of any adverse health outcome (HR, 0.87; 95% CI, 0.85 to 0.89), IHD (HR, 0.94; 95% CI, 0.91 to 0.97), stroke (HR, 0.85; 95% CI, 0.82 to 0.89), and diabetes-related hospitalization (HR, 0.73; 95% CI, 0.70 to 0.76).

**CONCLUSIONS:**

Participation in the CDMP was associated with improved long-term health outcomes among patients with T2DM. These findings support the importance of continuous, structured, primary care–based chronic disease management and provide empirical evidence for expanding and institutionalizing the CDMP within Korea’s National Health Insurance system.

## GRAPHICAL ABSTRACT

[Fig f2-epih-48-e2026023]

## Key Message

Although Korea’s Primary Care Chronic Disease Management Program (CDMP) was introduced to strengthen diabetes care, evidence on its long-term clinical effectiveness has been limited. In this nationwide study of patients with type 2 diabetes mellitus, participation in the CDMP was associated with reduced risks of preventable hospitalization, major cardiovascular and cerebrovascular events, and emergency department use over extended follow-up. These findings provide epidemiological evidence that structured, continuity-based primary care can improve long-term outcomes in chronic disease management and support the expansion and institutionalization of primary care–centered chronic disease programs within the National Health Insurance system.

## INTRODUCTION

Type 2 diabetes mellitus (T2DM) is a major global public health challenge and imposes a substantial burden on healthcare systems through avoidable hospitalizations and high complication rates [1]. The global prevalence of diabetes among adults aged 20–79 years is projected to reach 12.2% by 2045, suggesting that its impact will continue to intensify in the coming decades [2]. In Korea, diabetes accounts for 10.1% of the total disease burden, and T2DM accounts for nearly 87% of diabetes-related healthcare expenditures [3‑5].

Although T2DM can lead to serious complications, including cardiovascular, cerebrovascular, and renal diseases [6], sustained management can help prevent or control these complications through regular clinical follow-up, lifestyle modification, and early detection [7,8]. Evidence from Korea indicates that continuous care for patients with T2DM is associated with a significantly lower risk of complications than fragmented or intermittent care [9]. Findings from the United States Veterans Health Administration further indicate that uncoordinated or fragmented care is associated with higher hospitalization rates, reinforcing the importance of integrated primary care [10]. Collectively, these studies emphasize that continuity, coordination, and comprehensiveness in primary care are critical for improving outcomes among patients with chronic diseases such as diabetes.

Several countries, including the United Kingdom and Australia [11,12], have established structured national programs that strengthen chronic disease management in primary care through financial incentives and individualized care plans. Reflecting these initiatives, Korea implemented the Primary Care Chronic Disease Management Program (CDMP) in 2019 [13]. The program was introduced in January 2019 under the leadership of the Ministry of Health and Welfare to strengthen the role of local clinics in managing hypertension and diabetes by providing personalized education, counseling, lifestyle modification support, and support for medication adherence. As of December 31, 2022, 3,722 clinics across 109 regions nationwide had participated in the program, and 2,575 of these clinics had registered patients [14].

Initial evaluations of the CDMP have reported favorable outcomes, including improved blood pressure and glycemic control and enhanced patient self-management. Qualitative research indicated that participants expressed greater trust in their physicians and greater satisfaction with the accessibility and continuity of primary care services [15]. Quantitative analyses further showed that program participation was associated with higher medication adherence and better glycemic control, with approximately 79% of patients with diabetes achieving target glucose levels [16]. Despite these encouraging findings, most prior studies were conducted during the early phase of the program and focused on short-term or intermediate outcomes, such as reductions in glycated hemoglobin levels or improvements in medication adherence. Accordingly, evidence remains limited regarding the program’s long-term clinical benefits, particularly for major adverse clinical outcomes such as cardiovascular disease, cerebrovascular disease, and diabetes-related hospitalization. This study assessed the long-term effectiveness of Korea’s CDMP among patients with T2DM, focusing on preventable diabetes-related hospitalization and incident ischemic heart disease (IHD) and stroke.

## MATERIALS AND METHODS

### Study population

**Figure 1.**
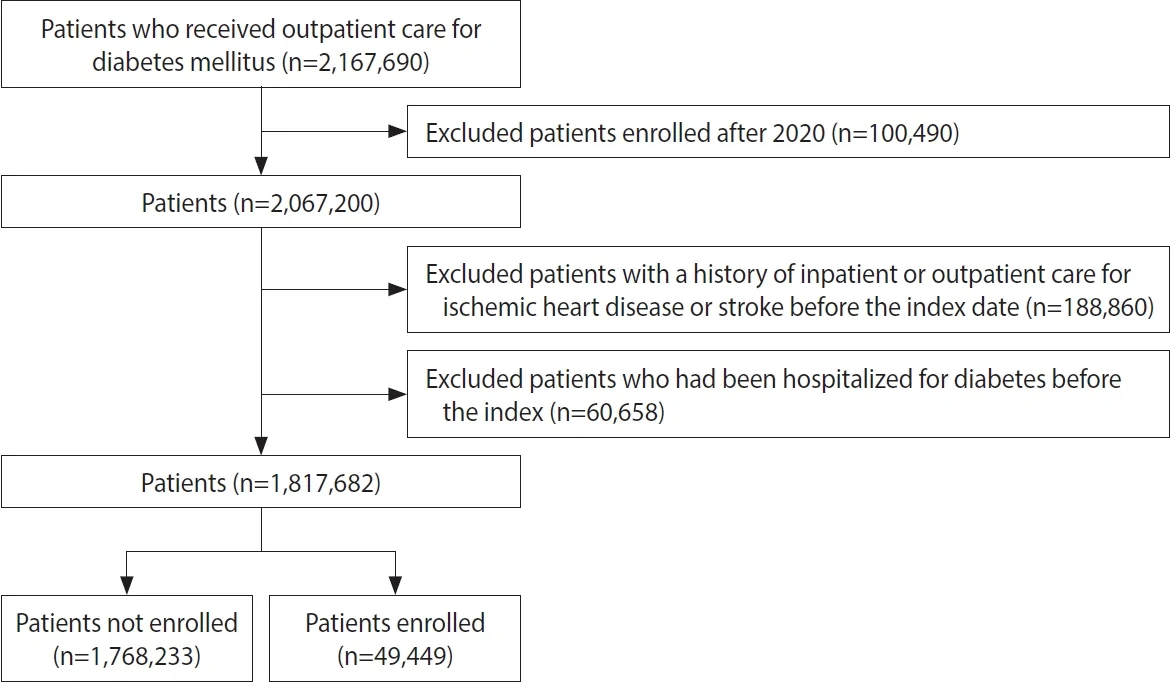
Flowchart of patient selection.

This nationwide population-based observational study used data from the National Health Insurance Claims Database (NHICD), which is maintained by the Health Insurance Review and Assessment Service of Korea (approval No. M20250428009). The NHICD includes nearly the entire Korean population covered by the National Health Insurance Program and is compiled primarily for reimbursement purposes [17]. The database contains detailed information on patient demographic characteristics, healthcare utilization, diagnostic codes based on the International Classification of Diseases, 10th revision (ICD-10), medical procedures, prescriptions, and providers. To evaluate the long-term effectiveness of the CDMP, adults aged ≥18 years who received outpatient care for T2DM at primary care clinics between January 2019 and December 2019, the initial implementation period, were identified. T2DM was defined as a principal or first secondary diagnosis coded as E11.x–E14.x according to the ICD-10. To include only patients who participated in the initial phase of the program, individuals newly enrolled after 2020 (n=100,490) and those with a prior history of inpatient or outpatient care for IHD or stroke, or a history of hospitalization for diabetes before the index date were excluded (Figure 1). The final cohort comprised 1,817,682 individuals.

### Variables

Three health outcomes were evaluated to assess the long-term effectiveness of the CDMP: preventable diabetes-related hospitalization, incident IHD, and incident stroke. Incident IHD (ICD-10 codes I20–I25) and stroke (ICD-10 codes I60–I64) were identified using primary and secondary diagnoses recorded in inpatient and outpatient settings [18,19]. Transient ischemic attack (ICD-10 code G45) was explicitly excluded from the stroke definition. For patients enrolled in the CDMP, the index date was defined as the date of program registration; for non-participants, the index date was defined as the date of the first outpatient visit for diabetes. Patients with a recorded code for the initial chronic disease assessment (IB011) were categorized as participants, whereas those without this code were classified as non-participants [13]. Each outcome was tracked from the index date through December 31, 2023, yielding a mean follow-up period of 4.4 years. Covariates included age, sex, type of health insurance, Charlson comorbidity index (CCI), and history of outpatient visits for diabetes before the index date. CCI and outpatient visit history were assessed during the 1-year period preceding the index date.

### Statistical analysis

Descriptive statistics were used to summarize baseline characteristics. Categorical variables are presented as frequencies and percentages, and continuous variables are presented as means and standard deviations (SDs). Between-group differences were assessed using the Student t-test for continuous variables and the chi-square test for categorical variables. Cox proportional hazards regression models were used to estimate hazard ratios (HRs) and 95% confidence intervals (CIs) for each health outcome after adjustment for age, sex, type of health insurance, CCI, and prior outpatient visits for diabetes. The proportional hazards assumption was assessed and satisfied. Furthermore, we conducted subgroup analyses stratified by diabetes status to evaluate whether the association between CDMP participation and complication risk differed. Patients were categorized into two subgroups: those with prevalent diabetes (diagnosed prior to the index date) and those with newly diagnosed diabetes (diagnosed during the study period). All analyses were conducted using SAS version 9.4 (SAS Institute Inc., Cary, NC, USA), with a 2-sided significance threshold of p-value <0.05.

### Ethics statement

The study protocol was approved by the Institutional Review Board of Kongju National University, which waived the requirement for informed consent (No. KNU_IRB_2025-031).

## RESULTS

Overall, 49,449 CDMP participants and 1,768,233 non-participants were included in this study (Table 1). The mean ages of the participant and non-participant groups were 62.2 years (SD, 11.3) and 61.5 years (SD, 12.7), respectively, and the between-group difference was significant. Sex distribution did not differ significantly between groups. National Health Insurance beneficiaries accounted for 90.0% of participants and 94.6% of non-participants, whereas Medical Aid recipients accounted for 10.0% and 5.4%, respectively. The proportions of participants with CCI scores of 0 and ≥3 were 16.6% and 25.6%, respectively; the corresponding proportions among non-participants were 26.3% and 18.1%, indicating a higher overall comorbidity burden in the participant group.

Among patients in the CDMP group in 2019, 15.6% experienced at least 1 of the health outcomes evaluated in this study, which was significantly lower than the proportion among non-participants (16.8%; Table 2). The rates of stroke and diabetes-related hospitalization were significantly lower in the CDMP group than in the non-CDMP group, whereas the incidence of IHD was similar between groups: 8.7% among non-participants and 8.6% among participants. In both groups, adverse health outcomes increased significantly with advancing age. Among individuals aged ≥65 years, the incidence of IHD was comparable between participants and non-participants, at 11.0% and 11.8%, respectively. In the CDMP group, the incidence of stroke was 4.7% in male and 4.9% in female; the corresponding proportions in the non-CDMP group were 5.1% and 5.6%, respectively, indicating consistently lower stroke incidence among both male and female participants. For diabetes-related hospitalization, the incidence among National Health Insurance beneficiaries was 3.5% in the CDMP group and 4.5% in the non-CDMP group; among Medical Aid beneficiaries, the corresponding rates were 6.3% and 8.3%, respectively, indicating lower hospitalization rates in the CDMP group across both insurance categories.

The risks of adverse health outcomes according to CDMP participation are presented in Table 3. Relative to the non-CDMP group, the CDMP group had a significantly lower risk of any of the evaluated adverse health outcomes (HR, 0.87; 95% CI, 0.85 to 0.89). In outcome-specific analyses, the risks of IHD and stroke were significantly lower in the CDMP group than in the non-CDMP group, with HRs of 0.94 (95% CI, 0.91 to 0.97) and 0.85 (95% CI, 0.82 to 0.89), respectively. Similarly, the risk of diabetes-related hospitalization was substantially lower in the CDMP group (HR, 0.73; 95% CI, 0.70 to 0.76). The risk of adverse health outcomes increased significantly with age; the risk of stroke was markedly higher among individuals aged ≥65 years than among those aged 18–44 years (HR, 10.22; 95% CI, 9.69 to 10.78). Female patients generally had lower risks of adverse outcomes than male patients, whereas Medical Aid beneficiaries had higher risks than National Health Insurance beneficiaries. A higher CCI was also associated with increased risks of adverse outcomes. In addition, patients with a history of outpatient visits for diabetes had significantly higher risks of IHD, stroke, and diabetes-related hospitalization than those without such a history.

## DISCUSSION

This nationwide cohort study evaluated the long-term association between participation in Korea’s CDMP and major clinical outcomes—specifically, diabetes-related hospitalization and incident IHD and stroke—among patients with T2DM. Whereas previous studies have primarily focused on the program’s early phase and intermediate markers, this study followed patients for a mean of 4.4 years and provides long-term clinical evidence that CDMP participation was associated with a significantly lower overall risk of major adverse health outcomes. These findings support the importance of continuous, structured, primary care–based management for improving long-term prognosis among individuals with T2DM.

The Korean healthcare system has historically assigned a relatively limited role to primary care, and many patients with chronic conditions use tertiary hospitals, contributing to an inefficient care delivery structure [13]. In addition, the average hospital stay among patients with diabetes in Korea is 16.5 days, considerably longer than the average of 8.1 days reported for member countries of the Organization for Economic Cooperation and Development, underscoring the need to strengthen primary care and improve the quality of primary care services. The CDMP was established not merely as an additional medical encounter but as a comprehensive system designed to strengthen the role of primary care clinics in chronic disease management. The program incorporates structured assessments, individualized education, lifestyle counseling, and medication-adherence support, resembling successful chronic care models such as the United Kingdom’s Quality and Outcomes Framework and Australia’s Chronic Disease Management initiative [11,12]. Consistent with these international programs, CDMP participation in this study was associated with a 13.3% lower risk of experiencing any adverse health outcome evaluated (HR, 0.87; 95% CI, 0.85 to 0.89).

Notably, the strongest association was observed for diabetes-related hospitalization: the CDMP group had a 27% lower risk than the non-CDMP group (HR, 0.73; 95% CI, 0.70 to 0.76). This finding suggests that sustained monitoring, early intervention, and patient education provided through the CDMP may help prevent acute metabolic decompensation and complications that lead to potentially avoidable hospitalizations. Consistent with these findings, a large Australian cohort study reported that greater longitudinal continuity of care was significantly associated with a lower risk of diabetes-related hospitalization. Patients with lower levels of general practitioner coverage had a substantially higher incidence of diabetes-related hospitalization (relative risk, 3.1; 95% CI, 2.0 to 4.9) [20]. Similarly, a Korean study evaluating a community-based primary care intervention demonstrated a significantly lower risk of hospitalization for diabetes-specific complications among patients with T2DM (HR, 0.76; 95% CI, 0.65 to 0.91) [21].

Together, these studies suggest that continuous access to optimized primary care is associated with fewer unnecessary hospital admissions and may contribute to healthcare cost savings. The risks of severe complications, including stroke and IHD, were also significantly lower in the CDMP group, by 15% (HR, 0.85; 95% CI, 0.82 to 0.89) and 6% (HR, 0.94; 95% CI, 0.91 to 0.97), respectively. The reduction in stroke risk was greater than that observed for IHD. This difference may indicate that the program’s emphasis on continuous blood pressure and glucose control, together with reinforced medication adherence, is particularly relevant to hypertension management, given that hypertension is more strongly associated with stroke than with IHD. The relationship between crude incidence and adjusted HRs for IHD also warrants consideration. Crude incidence differed only minimally between the CDMP group and the non-CDMP group, at 8.65% and 8.74%, respectively. However, the participant cohort was older on average and had a higher proportion of patients with severe comorbidities. In the unadjusted comparison, this higher baseline clinical risk may have masked the protective association between CDMP participation and IHD outcomes. After adjustment for these unfavorable baseline characteristics in the multivariable model, CDMP participation was associated with a significantly lower risk of IHD. This finding underscores the importance of rigorous case-mix adjustment when voluntary chronic care programs are evaluated using observational claims data. These findings are consistent with previous Korean research indicating that continuous primary clinic care is associated with a lower risk of cardiovascular disease hospitalization [22], as well as international evidence suggesting that structured chronic care programs may mitigate long-term cardiovascular risks [23].

In subgroup analyses stratified by diabetes status, the association between CDMP participation and reduced complication risk was observed only among patients with prevalent diabetes (HR, 0.84; 95% CI, 0.81 to 0.88); no statistically significant association was observed among patients with newly diagnosed diabetes during the study period (HR, 0.93; 95% CI, 0.84 to 1.02). This finding may reflect differences in baseline disease severity and cumulative exposure to hyperglycemia. Patients with newly diagnosed diabetes generally have shorter disease duration and lower absolute complication risk, because complications typically develop after prolonged metabolic dysregulation. Therefore, the relatively short follow-up period may have limited the ability to detect meaningful risk reductions in this subgroup. In contrast, patients with prevalent diabetes are more likely to have accumulated cardiometabolic risk and subclinical vascular damage, which may make them more likely to benefit from structured, continuous management interventions. The CDMP’s emphasis on sustained monitoring, medication adherence, and lifestyle modification may therefore confer greater absolute and relative benefits in this higher-risk population.

This study provides empirical evidence that participation in the CDMP is associated with lower long-term morbidity among patients with T2DM. This evidence supports expansion and institutionalization of the program within the National Health Insurance framework, moving it beyond pilot status toward permanent national policy. Continued promotion of the program is essential for Korea’s transition toward a community-based, primary care–centered healthcare system. However, this analysis also showed that socioeconomically or clinically vulnerable populations—including older adults aged ≥65 years, Medical Aid beneficiaries, and patients with a high comorbidity burden (CCI ≥3)—continued to have significantly higher health risks. Specifically, the risk of stroke among individuals aged ≥65 years was more than 10-fold higher than that among individuals 18 years to 44 years old (HR, 10.22; 95% CI, 9.69 to 10.78). This finding suggests that although the CDMP is associated with overall benefits, policymakers should consider more intensive resource allocation and tailored services for high-risk and vulnerable populations to improve equity in long-term chronic disease management outcomes.

This study has several limitations inherent to the use of the NHICD. First, the database lacks detailed clinical and behavioral information, such as glycated hemoglobin levels, blood pressure, smoking status, and disease duration. Consequently, residual confounding due to unmeasured disease severity and lifestyle factors cannot be ruled out. Although baseline medication patterns, such as insulin use or the number of oral hypoglycemic agents, can serve as surrogate markers of disease severity, these variables were not explicitly included as covariates in the study model. This decision was based on the methodological concern that prescription patterns in claims data often reflect not only clinical severity but also physician prescribing preferences and individual healthcare-utilization behaviors, which could introduce confounding by indication. To address baseline clinical risk, the CCI, a validated measure that includes diabetes-related complications, was used to capture overall comorbidity burden.

Second, program participation was defined dichotomously based on the initial assessment and care-plan development code (IB011). Although this code is a reliable indicator of initiation of the structured care pathway, it does not fully capture the intensity or continuity of participation, including the extent to which subsequent education and counseling components were delivered or sustained over the 1-year cycle. According to the official CDMP guidelines, the standard intervention protocol involves establishing a comprehensive care plan on a 1-year cycle, during which patients are eligible to receive up to 10 structured education and counseling sessions provided by physicians and care coordinators [13]. Although individual adherence may vary, this framework provides a baseline understanding of the intended intervention intensity. Therefore, the results reflect real-world effectiveness associated with program enrollment rather than effectiveness among patients who adhered to all intervention components. Although a dose-response analysis based on subsequent education billing codes was considered, this analysis was not performed to avoid important epidemiological biases. In the NHICD, the frequency of subsequent codes is confounded by intrinsic patient adherence and continuity of follow-up, introducing a substantial risk of adherence-related bias, including the healthy-adherer effect. Consequently, simple frequency-based measures serve as imperfect proxies for true intervention intensity. Because this limitation prevents detailed examination of potential dose-response relationships, future research should evaluate how the intensity and quality of these interventions influence long-term health outcomes.

Third, although a multivariable model was used to control for measured confounders, the voluntary nature of CDMP participation may have introduced healthy-user bias, because participants may have had stronger health-seeking behaviors than non-participants. Advanced causal inference methods, such as propensity score matching, could potentially mitigate this bias; however, multivariable Cox regression was selected to avoid substantial loss of data from the large non-CDMP group (>1.7 million) and to preserve the generalizability of the nationwide findings. This approach also allowed simultaneous assessment of the independent associations of age, sex, and type of health insurance with the study outcomes. Nevertheless, residual confounding due to unobserved factors remains possible, and the results should be interpreted with this limitation in mind.

This nationwide cohort study provides compelling evidence that participation in Korea’s CDMP is associated with improved long-term health outcomes, including lower risks of major cardiovascular and cerebrovascular events and diabetes-related hospitalization among patients with T2DM. These findings support efforts to strengthen continuous, comprehensive primary care delivery as part of sustainable chronic disease management policy in Korea.

## Figures and Tables

**Figure f2-epih-48-e2026023:**
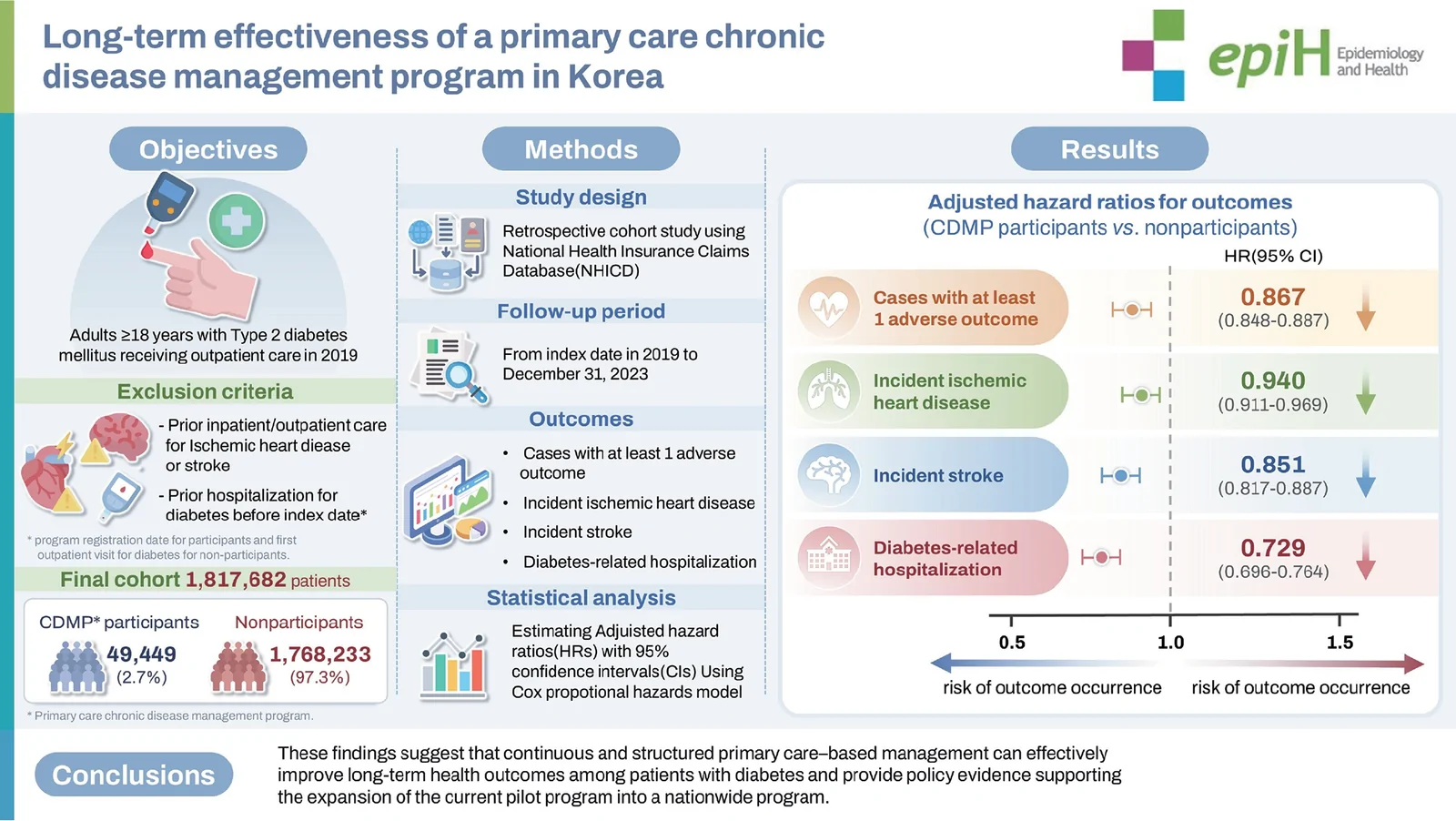


**Table 1.** General characteristics of the study population

Characteristics	Category	Total	CDMP	Non-CDMP	p-value
Total		1,817,682 (100)	49,449 (2.7)	1,768,233 (97.3)	
Age (yr)	Mean±SD	61.6±12.6	62.2±11.3	61.5±12.7	<0.001
	18–44	163,346 (9.0)	2,995 (6.1)	160,351 (9.1)	<0.001
	45–64	912,457 (50.2)	25,668 (51.9)	886,789 (50.2)	
	≥65	741,879 (40.8)	20,786 (42.0)	721,093 (40.8)	
Sex	Male	975,975 (53.7)	26,558 (53.7)	949,417 (53.7)	0.948
	Female	841,707 (46.3)	22,891 (46.3)	818,816 (46.3)	
Type of health insurance	National Health Insurance	1,718,000 (94.5)	44,511 (90.0)	1,673,489 (94.6)	<0.001
	Medical Aid	99,682 (5.5)	4,938 (10.0)	94,744 (5.4)	
CCI	0	473,897 (26.1)	8,195 (16.6)	465,702 (26.3)	<0.001
	1	604,374 (33.3)	15,681 (31.7)	588,693 (33.3)	
	2	406,730 (22.4)	12,938 (26.2)	393,792 (22.3)	
	≥3	332,681 (18.3)	12,635 (25.6)	320,046 (18.1)	
Previous outpatient care for DM	No	347,393 (19.1)	2,752 (5.6)	344,641 (19.5)	<0.001
	Yes	1,470,289 (80.9)	46,697 (94.4)	1,423,592 (80.5)	

Values are presented as number (%). CDMP, chronic disease management program; SD, standard deviation; CCI, Charlson comorbidity index; DM, diabetes mellitus.

**Table 2.** Comparison of adverse outcome incidence between CDMP participants and non-participants

Variables	Cases with at least 1 adverse outcome				Incident ischemic heart disease				Incident stroke				Diabetes-related hospitalization			
	Non-CDMP	p-value	CDMP	p-value	Non-CDMP	p-value	CDMP	p-value	Non-CDMP	p-value	CDMP	p-value	Non-CDMP	p-value	CDMP	p-value
Total	297,718 (16.8)		7,730 (15.6)		154,541 (8.7)		4,277 (8.6)		94,236 (5.3)		2,367 (4.8)		83,635 (4.7)		1,856 (3.8)	
Age (yr)		<0.001		<0.001		<0.001		<0.001		<0.001		<0.001		<0.001		<0.001
18–44	12,048 (7.5)		222 (7.4)		5,040 (3.1)		116 (3.9)		1,396 (0.9)		18 (0.6)		6,274 (3.9)		101 (3.4)	
45–64	115,996 (13.1)		3,220 (12.5)		64,539 (7.3)		1,870 (7.3)		27,240 (3.1)		760 (3.0)		35,137 (4.0)		858 (3.3)	
≥65	169,674 (25.5)		4,288 (20.6)		84,962 (11.8)		2,291 (11.0)		65,600 (9.1)		1,589 (7.6)		42,224 (5.9)		897 (4.3)	
Sex		<0.001		0.003		<0.001		<0.001		<0.001		0.326		0.166		0.017
Male	160,831 (16.9)		4,270 (16.1)		86,431 (9.1)		2,412 (9.1)		48,668 (5.1)		1,248 (4.7)		44,711 (4.7)		1,047 (3.9)	
Female	136,887 (16.7)		3,460 (15.1)		68,110 (8.3)		1,865 (8.2)		45,568 (5.6)		1,119 (4.9)		38,924 (4.8)		809 (3.5)	
Type of insurance		<0.001		<0.001		<0.001		<0.001		<0.001		<0.001		<0.001		<0.001
National Health Insurance	273,880 (16.4)		6,593 (14.8)		143,530 (8.6)		3,698 (8.3)		85,741 (5.1)		1,974 (4.4)		75,741 (4.5)		1,547 (3.5)	
Medical Aid	23,838 (25.2)		1,137 (23.0)		11,011 (11.6)		579 (11.7)		8,495 (9.0)		393 (8.0)		7,894 (8.3)		309 (6.3)	
CCI		<0.001		<0.001		<0.001		<0.001		<0.001		<0.001		<0.001		<0.001
0	62,777 (13.5)		971 (11.9)		29,994 (6.4)		490 (6.0)		19,862 (4.3)		301 (3.7)		19,535 (4.2)		261 (3.2)	
1	89,460 (15.2)		2,069 (13.2)		45,648 (7.8)		1,149 (7.3)		27,624 (4.7)		594 (3.8)		25,748 (4.4)		499 (3.2)	
2	72,180 (18.3)		2,081 (16.1)		38,409 (9.8)		1,120 (8.7)		22,723 (5.8)		658 (5.1)		19,562 (5.0)		513 (4.0)	
≥3	73,301 (22.9)		2,609 (20.7)		40,490 (12.7)		1,518 (12.0)		24,027 (7.5)		814 (6.4)		18,790 (5.9)		583 (4.6)	
Previous outpatient care for DM		<0.001		0.005		<0.001		0.529		<0.001		0.628		<0.001		<0.001
No	43,431 (12.6)		378 (13.7)		24,910 (7.2)		229 (8.3)		13,688 (4.0)		137 (5.0)		8,369 (2.4)		44 (1.6)	
Yes	254,287 (17.9)		7,352 (15.7)		129,631 (9.1)		4,048 (8.7)		80,548 (5.7)		2,230 (4.8)		75,266 (5.3)		1,812 (3.9)	

CDMP, chronic disease management program; CCI, Charlson comorbidity index; DM, diabetes mellitus.

**Table 3.** Cox proportional hazards model for the incidence of adverse outcomes

Variables	Category	Cases with at least 1 adverse outcome	p-value	Incident ischemic heart disease	p-value	Incident stroke		Diabetes-related hospitalization	p-value
Group	Non-CDMP	Reference		Reference		Reference		Reference	
	CDMP	0.87 (0.85, 0.89)	<0.001	0.94 (0.91, 0.97)	<0.001	0.85 (0.82, 0.89)	<0.001	0.73 (0.70, 0.76)	<0.001
Age (yr)	18–44	Reference		Reference		Reference		Reference	
	45–64	1.68 (1.64, 1.71)	<0.001	2.23 (2.17, 2.29)	<0.001	3.42 (3.24, 3.61)	<0.001	0.89 (0.87, 0.92)	<0.001
	≥65	3.10 (3.04, 3.16)	<0.001	3.59 (3.49, 3.69)	<0.001	10.22 (9.69, 10.78)	<0.001	1.26 (1.22, 1.29)	<0.001
Sex	Male	Reference		Reference		Reference		Reference	
	Female	0.83 (0.82, 0.83)	<0.001	0.77 (0.77, 0.78)	<0.001	0.83 (0.82, 0.85)	<0.001	0.92 (0.91, 0.93)	<0.001
Type of health insurance	National Health Insurance	Reference		Reference		Reference		Reference	
	Medical Aid	1.43 (1.41, 1.44)	<0.001	1.21 (1.19, 1.23)	<0.001	1.51 (1.47, 1.54)	<0.001	1.75 (1.71, 1.79)	<0.001
CCI	0	Reference		Reference		Reference		Reference	
	1	1.08 (1.07, 1.09)	<0.001	1.17 (1.16, 1.19)	<0.001	1.02 (1.00, 1.04)	0.042	0.98 (0.96, 1.00)	0.033
	2	1.25 (1.24, 1.27)	<0.001	1.42 (1.40, 1.44)	<0.001	1.13 (1.11, 1.16)	<0.001	1.08 (1.06, 1.10)	<0.001
	≥3	1.49 (1.48, 1.51)	<0.001	1.77 (1.74, 1.79)	<0.001	1.31 (1.28, 1.33)	<0.001	1.22 (1.20, 1.25)	<0.001
Previous outpatient care for DM	No	Reference		Reference		Reference		Reference	
	Yes	1.20 (1.19, 1.21)	<0.001	1.04 (1.03, 1.06)	<0.001	1.11 (1.09, 1.13)	<0.001	1.98 (1.93, 2.02)	<0.001

Values are presented as hazard ratio (95% confidence interval). CDMP, chronic disease management program; CCI, Charlson comorbidity index; DM, diabetes mellitus.
